# Subcortical Shape Changes, Hippocampal Atrophy and Cortical Thinning in Future Alzheimer's Disease Patients

**DOI:** 10.3389/fnagi.2017.00038

**Published:** 2017-03-07

**Authors:** Andrea M. Kälin, Min T. M. Park, M. Mallar Chakravarty, Jason P. Lerch, Lars Michels, Clemens Schroeder, Sarah D. Broicher, Spyros Kollias, Roger M. Nitsch, Anton F. Gietl, Paul G. Unschuld, Christoph Hock, Sandra E. Leh

**Affiliations:** ^1^Institute for Regenerative Medicine, University of ZurichSchlieren, Switzerland; ^2^Cerebral Imaging Centre, Douglas Mental Health University InstituteMontreal, QC, Canada; ^3^Schulich School of Medicine and Dentistry, Western UniversityLondon, ON, Canada; ^4^Departments of Psychiatry and Biological and Biomedical Engineering, McGill UniversityMontreal, QC, Canada; ^5^The Hospital for Sick ChildrenToronto, ON, Canada; ^6^Department of Medical Biophysics, The University of TorontoToronto, ON, Canada; ^7^Clinic of Neuroradiology, University Hospital Zurich, University of ZurichZurich, Switzerland; ^8^Center for MR Research, University Children's Hospital ZurichZurich, Switzerland; ^9^Neuropsychology Unit, Department of Neurology, University Hospital ZurichZurich, Switzerland

**Keywords:** Alzheimer's disease, mild cognitive impairment, cortical thickness, subcortical shape analysis, hippocampal subfields

## Abstract

Efficacy of future treatments depends on biomarkers identifying patients with mild cognitive impairment at highest risk for transitioning to Alzheimer's disease. Here, we applied recently developed analysis techniques to investigate cross-sectional differences in subcortical shape and volume alterations in patients with stable mild cognitive impairment (MCI) (*n* = 23, age range 59–82, 47.8% female), future converters at baseline (*n* = 10, age range 66–84, 90% female) and at time of conversion (age range 68–87) compared to group-wise age and gender matched healthy control subjects (*n* = 23, age range 61–81, 47.8% female; *n* = 10, age range 66–82, 80% female; *n* = 10, age range 68–82, 70% female). Additionally, we studied cortical thinning and global and local measures of hippocampal atrophy as known key imaging markers for Alzheimer's disease. Apart from bilateral striatal volume reductions, no morphometric alterations were found in cognitively stable patients. In contrast, we identified shape alterations in striatal and thalamic regions in future converters at baseline and at time of conversion. These shape alterations were paralleled by Alzheimer's disease like patterns of left hemispheric morphometric changes (cortical thinning in medial temporal regions, hippocampal total and subfield atrophy) in future converters at baseline with progression to similar right hemispheric alterations at time of conversion. Additionally, receiver operating characteristic curve analysis indicated that subcortical shape alterations may outperform hippocampal volume in identifying future converters at baseline. These results further confirm the key role of early cortical thinning and hippocampal atrophy in the early detection of Alzheimer's disease. But first and foremost, and by distinguishing future converters but not patients with stable cognitive abilities from cognitively normal subjects, our results support the value of early subcortical shape alterations and reduced hippocampal subfield volumes as potential markers for the early detection of Alzheimer's disease.

## Introduction

Recent findings have demonstrated that beta-amyloid can be effectively removed from the brain, which may have a beneficial effect on cognitive function (Sevigny et al., 2016). Following the concept of the amyloid-hypothesis, Alzheimer's disease (AD) treatment methods that are currently under investigation may be most efficacious in preclinical or early disease stages (Golde et al., 2011; Sperling et al., 2011; Sevigny et al., 2016), referred to as mild cognitive impairment (MCI). Here, MCI is used as umbrella term covering subjects with stable cognitive impairment without progression to AD as well as subjects with worsening of cognitive impairment and progression to AD. Considering that only the latter will benefit from potential treatment methods, the establishment and validation of biomarkers that accurately identify future converters to AD among individuals with MCI is crucial.

Structural magnetic resonance imaging (MRI) allows the quantification of brain atrophy and represents a key imaging marker for the early detection of AD. Specifically, reduced gray matter volume in medial temporal lobe regions including the hippocampal formation may precede the clinical onset of AD by 10 years (Tondelli et al., 2012). Consequently, the applicability of hippocampal subfield segmentation in clinical populations has gained increasing attention, with different studies providing evidence for predominant cornu ammonis (CA)1 and subiculum atrophy in MCI (Atienza et al., 2011; Pluta et al., 2012; Yushkevich et al., 2015a) and AD (Frisoni et al., 2008; Mueller and Weiner, 2009; Wisse et al., 2014a). Cortical thickness analysis constitutes another widely accepted approach to measure gray matter atrophy in AD, and cortical thinning has been found in MCI and AD (Lerch et al., 2008; Liao et al., 2014), in late compared to early amnestic MCI (Ye et al., 2014), and in future converters (Bakkour et al., 2009; Julkunen et al., 2010; Li et al., 2012; Liao et al., 2014). Although gray matter alterations assessed by MRI represent valuable biomarkers for AD, the appropriate characterization of the progressing pattern of AD pathology across the brain (Braak and Braak, 1991a,b) might require the consideration of multiple structures as well as standardized procedures/analysis methods. Additionally, as results from hippocampal subfield analyses have indicated, more local information about different structures might further contribute to the characterization of AD-typical patterns of morphometric alterations.

Despite evidence for subcortical amyloid and neurofibrillary tangle formation in AD (Braak and Braak, 1990, 1991b), MRI research has drawn its attention to AD related subcortical structure changes only recently. Advanced segmentation techniques now permit the quantification of subcortical volumes and provide the basis for subcortical shape analysis. Although volume loss and/or shape alterations in the thalamus (Zarei et al., 2010; Roh et al., 2011; Stepan-Buksakowska et al., 2014), putamen (Roh et al., 2011; Cho et al., 2014; De Jong et al., 2014) and caudate nucleus (Madsen et al., 2010; Roh et al., 2011; Cho et al., 2014) have been identified in AD, little is known about subcortical volumetric and shape differences in MCI in general, and in future converters in particular. Given the connectivity of the thalamus and striatum to other AD-relevant structures, such as the hippocampus (Zarei et al., 2010), alterations in these structures may be of high value for the early detection of AD.

Here, we investigated subcortical volume and shape alterations as well as cortical thickness and volumes in the hippocampus and its subfields in MCI with stable cognitive abilities compared to healthy control subjects (HC) as well as in MCI with future conversion to AD at their baseline and conversion timepoints. In this manner we were able to elucidate the potential value of subcortical shape measures and their ability to improve the identification of future converters to AD. Further, we use cortical and hippocampal measures to demonstrate anatomical trajectories of the subjects under study, thereby confirming their role in the early detection of AD.

We expected stable MCI subjects to show no morphometric alterations typically related to AD, such as hippocampal atrophy or cortical thinning when compared with HC. In contrast, we expected morphometric alterations in accordance with known AD related histopathological processes in future converters at baseline and—more pronounced—at time of conversion. Specifically, and based on the pattern of AD related neurofibrillary tangle formation (Braak and Braak, 1990, 1991a,b), we expected volume reductions in the hippocampus (CA1, subiculum), the thalamus (anterior subregions) and the striatum in future converters at baseline and at time of conversion. Further confirming AD related neurodegenerative patterns (Lerch et al., 2008; Bakkour et al., 2009; Dickerson et al., 2009; Liao et al., 2014; Ye et al., 2014) in future converters at baseline and at time of conversion, we additionally expected cortical thinning in mediotemporal as well as lateral parietal and frontal regions and in the limbic system. Most importantly, and considering that subcortical volume reductions as well as possible secondary downstream effects may lead to thalamic and striatal shape alterations, we expected shape alterations in these structures to occur in both, future converters at basline and at time of conversion.

## Materials and methods

### Participants

We selected participants from different pre-existing longitudinal cohorts at the Memory Clinic of the Division of Psychiatry Research and Psychogeriatric Medicine, University of Zurich. Briefly, participants were recruited from the outpatient population of the Memory Clinic or through advertisements in the local media. HC were retrospectively and additionally recruited through inquiries of caregivers or relatives of the patients. MCI was diagnosed according to Winblad et al. (2004), based on performance in multiple tests covering the following cognitive domains: episodic memory, executive function, attention/psychomotor processing speed, language and visual-constructive abilities. Impairment was defined if at least one score per domain was 1.5 SD below group means provided by test-specific normative data. Conversion to dementia was diagnosed when clinical work up indicated progression and significant impairment in activities of daily living. This was assessed by a multidisciplinary team under the supervision of an experienced psychiatrist.

For the present study, inclusion criteria for MCI subjects were: amnestic MCI (single or multiple domain) diagnosis and availability of MRI data at baseline and follow-up. Exclusion criteria were: left-handedness, significant medication or drug abuse as well as clinically significant neurological and psychiatric or internal disease that may affect cognition, MRI findings of infarction or other focal lesions, multiple lacunes or lacunes in critical memory structures. A total of 33 baselines from subjects with amnestic MCI were considered for the present study. The population was stratified into subjects with stable cognitive abilities during an approximately 2-year follow-up (MCI-S, *n* = 23), and subjects with future cognitive worsening and conversion to probable AD (MCI-CB, *n* = 10) within a 2-year time frame during follow-up. Additionally, data from the MCI-CB group at time of conversion was obtained (MCI-CC, *n* = 10). Inclusion criteria for HC were: stable cognitive health ascertained by clinical work up and neuropsychological testing during an approximately 2-year follow-up. Exclusion criteria were: MRI exclusion criteria, left-handedness, evidence for abuse of alcohol and drugs, psychiatric, neurological or significant other system diseases. Three groups of HC were identified for group wise age and gender matching with MCI-S, MCI-CB, and MCI-CC, and MRI data was acquired following the description in section Magnetic Resonance Image Acquisition. The final demographic details are presented in Table 1. This study was approved by the cantonal ethics committee of canton Zurich, Switzerland, in accordance with the Helsinki Declaration. All participants provided written informed consent prior to study inclusion.

**Table 1:** **Demographic information and cognitive measures for patient and control groups**.

	**Group 1**		***p***	**Group 2**		***p***	**Group 3**		***p***
	**HC**	**MCI-S**		**HC**	**MCI-CB**		**HC**	**MCI-CC**	
N	23	23		10	10		10	10	
Age, years	70.96 5.78	71.22 6.54	0.887	75.90 5.99	76.20 6.25	0.914	77.40 5.34	78.00 6.24	0.778
Education, years	14.35 2.69	14.17 3.01	0.672	14.70 4.03	13.40 3.40	0.376	13.30 2.41	13.40 3.41	0.804
Gender, M/F	12/11	12/11	1.000	2/8	1/9	1.000	3/7	1/9	0.582
FU time, months^a^	46.91 33.89	25.91 8.41	N/A	39.50 32.35	20.80 4.98	N/A	48.10 33.22	N/A	N/A
MMSE, /30	29.83 0.38	28.09 1.67	0.000^*^	29.30 0.95	26.60 1.95	0.000^*^	29.30 1.06	23.50 2.55	0.000^*^
aMCI s/m	N/A	0/23	N/A	N/A	3/7	N/A	N/A	N/A	N/A
***EPISODIC MEMORY***									
VLMT learning	57.96 10.96	40.39 8.41	0.000^*^	54.80 12.12	27.86 6.49	0.000^*^	57.10 12.17	24.90 5.74	0.000^*^
VLMT recall	12.39 2.25	6.19 3.47	0.000^*^	11.50 2.50	1.71 1.49	0.000^*^	11.20 2.86	0.79 1.69	0.000^*^
***EXECUTIVE FUNCTIONS***									
Animal fluency	25.48 5.55	19.04 5.11	0.000^*^	22.10 4.45	15.20 3.85	0.002^*^	23.40 3.80	11.80 4.31	0.000^*^
Letter fluency	33.09 11.33	22.78 11.00	0.003^*^	29.90 7.76	25.20 10.01	0.259	32.50 8.38	24.70 9.79	0.072
DigitSpan bw	6.87 1.79	5.13 1.68	0.001^*^	5.50 1.08	5.40 1.17	0.976	5.70 1.89	5.30 1.49	0.606
***PSYCHOMOTOR SPEED/ATTENTION***									
TMT A	36.65 8.20	50.87 23.57	0.011^*^	44.50 7.05	43.90 10.82	0.885	39.50 12.32	58.50 20.11	0.020^*^
TMT B	87.52 25.80	156.39 60.8	0.000^*^	111.60 36.7	162.50 75.8	0.079	101.3 37.28	N/A^b^	N/A^b^
***LANGUAGE***									
BNT	14.74 0.45	13.61 1.15	0.000^*^	14.90 0.32	13.70 1.25	0.015^*^	14.80 0.42	12.80 1.47	0.000^*^

*Values are means and standard deviations if not specified otherwise. HC, healthy control subjects; MCI-S, stable mild cognitive impairment at baseline; MCI-CB, mild cognitive impairment converters at baseline; MCI-CC, mild cognitive impairment converters at time of conversion; M/F, male/female; FU, follow-up; MMSE, Mini Mental State Examination; aMCI s/m, amnestic mild cognitive impairment single domain (s), multiple domain (d); VLMT, Verbaler Lern—und Merkfähigkeitstest; bw, backward; TMT, Trail Making Test; BNT, Boson Naming Test; N/A, not applicable*.

* *Significant p value test by ANOVA (normally distributed data), Mann U Whitney (not normally distributed data), and Pearson's X^2^ test (categorical variables)*.

a *Time from baseline MRI to the time of Alzheimer's disease diagnosis for MCI-CB; time from baseline MRI to the last available visit for MCI-S; time from the first HC diagnosis to the last available visit for HC*.

b *high percentage of missing values due to the subjects' impairment did not allow statistical analyses*.

### Magnetic resonance image acquisition

All (MRI) were performed on the same 1.5 Tesla Phillips Achieva scanner using an 8-element head coil. Whole-brain high-resolution 3D T_1_-weighted structural data was obtained by using the following scanning parameters: 166 slices, repetition time: 6.9 ms, echo time: 3.2 ms, flip angle: 8°, field of view: 240 × 240 × 166 mm (anterior-posterior, foot-head, right-left), slice thickness: 1 mm, total scan time: 15 min.

### Image processing: subcortical structures and hippocampus

Segmentation of the striatum, thalamus and thalamic nuclei was performed using a recently developed label-fusion-based segmentation method that had previously proven its high accuracy (Chakravarty et al., 2013). Briefly, the MAGeT-Brain algorithm applies multiple automatically generated templates from a single atlas derived from manually segmented serial histological data comprising 108 basal ganglia and thalamic structures as defined using three different references (Schaltenbrand and Wahren, 1977; Hirai and Jones, 1989; Gloor, 1997). We used two of the segmentations produced from the MAGeT-Brain pipeline, the first are the whole striatum (caudate and putamen) and thalamus, and the second are the thalamic subnuclei as per the Hirai and Jones definitions (1989). The thalamus was segmented into pulvinar-, anterior-, and central nuclei and lateral dorsal-, lateral posterior-, medial dorsal nuclei, ventral anterior nuclei (VA), ventral lateral nuclei (VL), ventral posterior nuclei (VP) and lateral geniculate nucleus (LGN) and medial geniculate nucleus (MGN) as per the Hirai and Jones (1989) nomenclature. Segmentation of the hippocampus and its subfields was performed using five high-resolution atlases developed and validated for use with MAGeT-Brain (Winterburn et al., 2013; Pipitone et al., 2014). The hippocampus was segmented into cornu ammonis (CA) 1, CA2-CA3, CA4/Dentate gyrus, strata radiatum/lacunosum/moleculare, and subiculum.

### Surface-based shape analyses

Striatal and thalamic shape analysis was performed by using an adapted surface-based methodology (Magon et al., 2014; Raznahan et al., 2014; Shaw et al., 2015). Briefly, surface-based representations of the striatum and thalamus were defined on the input atlas. The nonlinear portions of the transformations that map each subject to the input template were concatenated and then averaged to limit the effects of noise and error and to increase precision and accuracy. Next, the dot product between the nonlinear deformation vector (of the inverse of the averaged atlas-to-subject transformation) and the surface normal at each vertex (a unit vector describing the direction perpendicular to the surface) was estimated. This measure provides an estimate of the local measure of inward or outward displacement along the normal. Then, surface-area values were blurred using a 5 mm surface-based diffusion smoothing kernel (Raznahan et al., 2014; Chakravarty et al., 2015). Resulting inward and outward displacements (measured in millimeters) were estimated relative to a detailed subcortical atlas previously described (Chakravarty et al., 2015). An inward displacement (contraction) represents a surface that is deformed inwards relative to the model that we were using and vice-versa for the outward displacement (expansion).

### Cortical thickness analyses

Cortical thickness was estimated by using the automated CIVET pipeline (version 1.1.10; Montreal Neurological Institute at McGill University, Montreal, Quebec, Canada). Briefly, the native images were linearly registered to the symmetric ICBM 152 template (Collins et al., 1994; Mazziotta et al., 2001). Intensity nonuniformities were corrected using the N3 algorithm (Sled et al., 1998). The skull was removed (Smith, 2002), and brain tissue was segmented into white matter, gray matter, cerebrospinal fluid (CSF) using the Intensity—Normalized Stereotaxic Environment for Classification of Tissues (INSECT) algorithm (Zijdenbos et al., 1998; Tohka et al., 2004). Deformable models were used to construct the inner white matter surface and gray matter-CSF interface in both hemispheres (Kim et al., 2005) revealing 40,962 vertex points at each surface. Cortical thickness was then measured as the distance, in millimeters, between each vertex point at the inner and the corresponding point at the outer surface using the method proposed by Lerch and Evans (2005). The cortical thickness maps were blurred using a 20 mm diffusion smoothing kernel to increase signal-to-noise ratio and statistical power.

### Intracranial volume

The comparison of gray matter volumes across groups requires taking into account interindividual variability in brain morphology. Values of intracranial volume (ICV) indicate premorbid brain volume, and thus are often used to adjust volumes for subsequent volume analyses. When examining volume reductions in neurodegenerative disease, considering ICV allows for estimation of atrophy caused by neurodegenerative mechanisms rather than by interindividual differences in head size and brain morphology.

In the present work, the FreeSurfer pipeline (version 5.1.0) was used to calculate total intracranial volume (eTIV) representing an estimate for ICV as described in Buckner et al. (2004). Briefly, each individual is registered to an atlas template. The Atlas Scaling Factor obtained by this transformation represents the whole-brain volume adjustment that is required to match each individual to the atlas template and is thus used to automatically generate eTIV. This automated method has been shown to be equivalent to manual correction (Buckner et al., 2004) and has previously been used for normalization in several AD studies (Westman et al., 2011, 2012, 2013).

### Statistical analyses

Group comparisons of demographic and conitive data were applied using analysis of variance (ANOVA) or Mann-Whitney *U*-Test. Pearson's chi-square test was used for categorical variables. Tests were performed with a significance level of *p* < 0.05. Between-group differences in volumetric raw data (MCI-S, MCI-CB and MCI-CC vs. matched HC) were examined by including age and gender as covariates in the multiple linear regression models. These analyses were repeated by using volumes relative to eTIV (volume/eTIV^*^100) in order to adjust volumes for differences in head size. *P*-values resulting from volume analyses were adjusted for multiple testing by using Bonferroni-Holm correction (level of significance for hippocampus, striatum and thalamus starting with *p* < 0.05/2; level of significance for hippocampal subfields starting with *p* < 0.05/10). The same models were performed for investigating between-group shape and cortical thickness differences. Vertex-wise analyses results are reported on a *q*-value corrected for multiple testing, using a false discovery rate (FDR) of 10% as in previous publications in our group (Wheeler et al., 2013; Janes et al., 2014). Receiver operating characteristic (ROC) curves were computed to evaluate and compare the accuracy of striatal and thalamic shape alterations, and of hippocampus total volumes as established AD imaging marker for discriminating HC from MCI-CB. ROC curves are produced by plotting the true positive rate (sensitivity) against the false positive rate (1-specificity) for different thresholds. The area under the curve (AUC) is then calculated and provides information about the ability of the morphometric data to discriminate between patients and HC. AUC values of 1.0 indicate perfect discriminative abilities; values of < 0.6 indicate poor discriminative abilities. For these analyses, between group t statistics from comparison between HC and MCI-CB were used. More precisely, for all vertices whose *t*-values constituted a local minimum (or maximum), the average of the displacement values in mm was computed for each subject. This was done separately for each structure and hemisphere. Statistical analyses were performed with IBM SPSS statistics 21 and RMINC package (R for Medical Imaging NetCDF; https://github.com/Mouse-Imaging-Centre/RMINC), an image analysis software library developed for the R statistical environment (http://www.r-project.org).

## Results

Descriptive statistics for demographic information and cognitive data is listed in Table 1.

### Volumetric analyses in MCI-S

**Figure 1:**
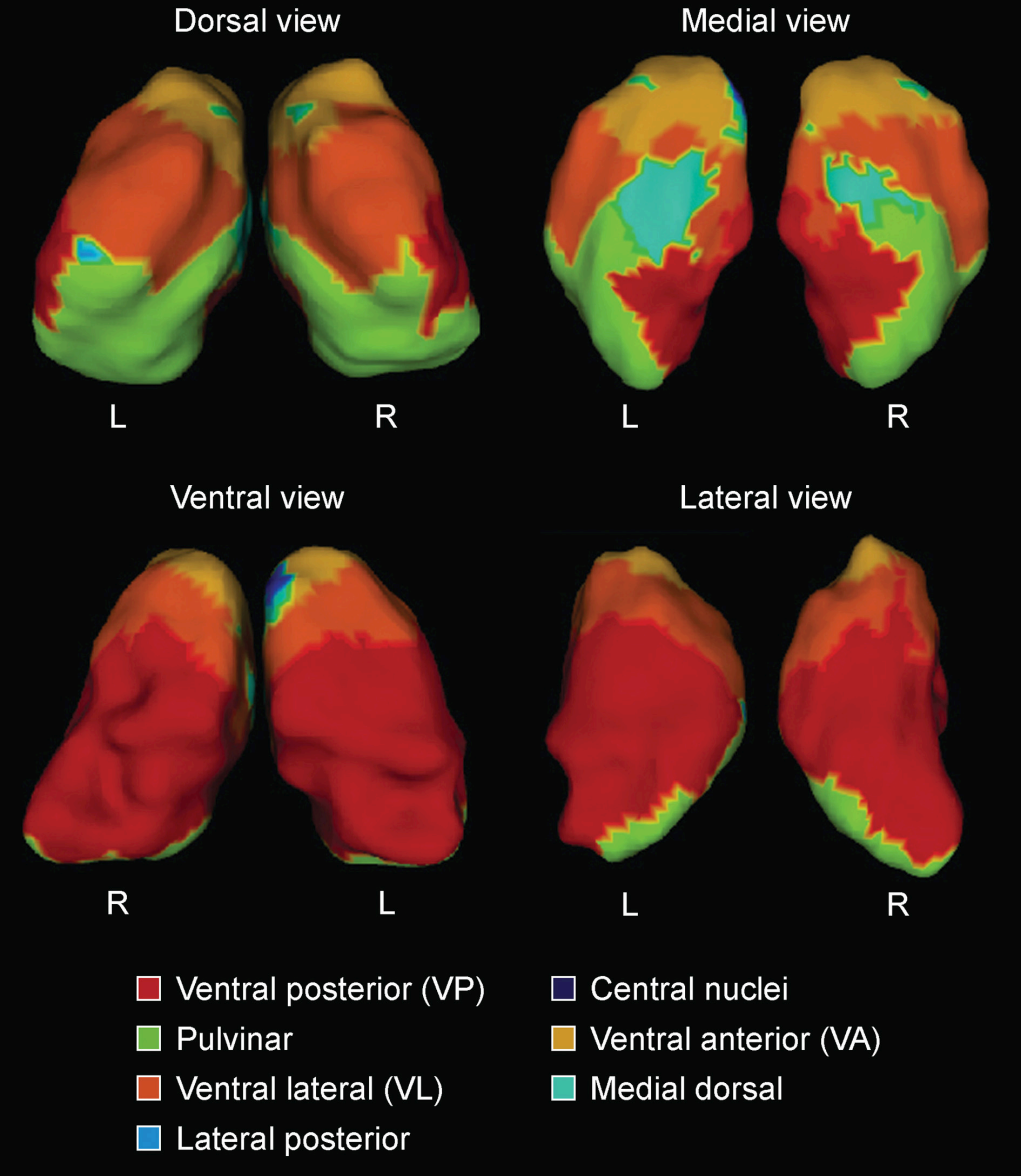
**Surface labels for automated segmentation of thalamic subnuclei (L = left hemisphere; R = right hemisphere), based on expert neuroanatomical labeling of serial histology (Chakravarty et al., 2006)**. Reprinted from Journal of Alzheimer's Disease 49(1) Leh SE, Kälin AM, Schroeder C, Park MT, Chakravarty MM, Freund P, Gietl AF, Riese F, Kollias S, Hock C, Michels L “Volumetric and shape analysis of the thalamus and striatum in amnestic mild cognitive impairment” 237–249, Copyright 2015, with permission from IOS Press.

**Figure 2:**
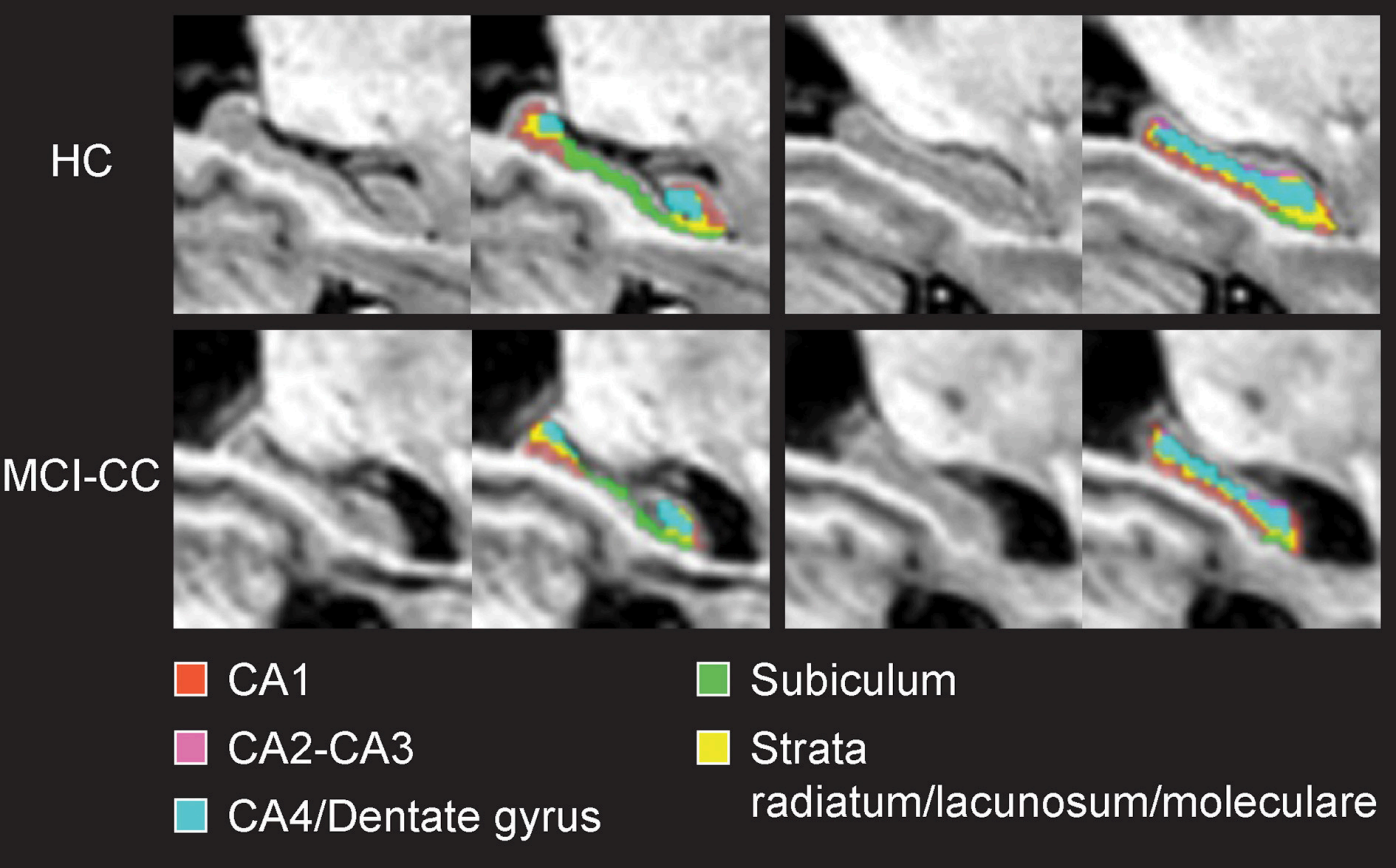
**Coronal views of the hippocampus and hippocampal subfields in magnetic resonance images from a healthy control subject (HC) and a mild cognitive impairment converter subject at time of conversion (MCI-CC) of the present study**.

Apart from reduced bilateral striatal volumes, and when analyzing volumes relative to eTIV, there were no volume differences in any of the investigated structures in MCI-S when compared with HC (see Table 2). Significance and *p*-values were similar when using raw volumes instead of volumes relative to eTIV (see Table 3). A segmentation map of the thalamus is shown in Figure 1, and of the hippocampus in Figure 2.

**Table 2:** **Volume sizes relative to eTIV for patient and control groups**.

	**Group 1**		***p***	**Group 2**		***p***	**Group 3**		***p***
	**HC**	**MCI-S**		**HC**	**MCI-CB**		**HC**	**MCI-CC**	
eTIV, cm^3^	1586.33 177.35	1546.71 155.10	0.424	1499.99 155.00	1498.05 133.58	0.976	1487.72 132.59	1507.66 133.52	0.741
Hippocampus total, left	0.14206 0.01670	0.13915 0.02510	0.670	0.14995 0.01635	0.11236 0.013455	0.000^*^	0.14493 0.00780	0.10656 0.01278	0.000^*^
Subiculum	0.02103 0.00250	0.01972 0.00379	0.162	0.02210 0.00243	0.01650 0.00230	0.000^*^	0.02060 0.00222	0.01579 0.00260	0.000^*^
CA1	0.04545 0.00509	0.04568 0.00773	0.872	0.04834 0.00525	0.03648 0.00436	0.000^*^	0.04651 0.00348	0.03587 0.00509	0.000^*^
CA2-CA3	0.00851 0.00180	0.00849 0.00200	0.966	0.00882 0.00184	0.00752 0.00154	0.088	0.00904 0.00088	0.00659 0.00162	0.001^*^
CA4/Dentate gyrus	0.03399 0.00460	0.03264 0.00611	0.415	0.03594 0.00464	0.02756 0.00380	0.000^*^	0.03500 0.00282	0.02583 0.00354	0.000^*^
Strata	0.03308 0.00449	0.03262 0.00682	0.821	0.03475 0.00417	0.02428 0.00335	0.000^*^	0.03377 0.00247	0.02249 0.00255	0.000^*^
Hippocampus total, right	0.14140 0.01557	0.14454 0.01796	0.495	0.14991 0.01238	0.12429 0.02027	0.001^*^	0.14467 0.00825	0.11796 0.01651	0.000^*^
Subiculum	0.01936 0.00235	0.01907 0.00326	0.753	0.02079 0.00254	0.01677 0.00321	0.005^*^	0.01998 0.00258	0.01590 0.00277	0.001^*^
CA1	0.04698 0.00538	0.04862 0.00607	0.335	0.05002 0.00413	0.04260 0.00668	0.005^*^	0.04862 0.00335	0.04165 0.00626	0.005^*^
CA2-CA3	0.00926 0.00125	0.00958 0.00162	0.449	0.00961 0.00135	0.00848 0.00195	0.068	0.00933 0.00179	0.00750 0.00210	0.044^*^
CA4/Dentate gyrus	0.03396 0.00414	0.03464 0.00396	0.519	0.03560 0.00418	0.02973 0.00471	0.002^*^	0.03426 0.00261	0.02795 0.00360	0.000^*^
Strata	0.03184 0.00438	0.03263 0.00516	0.534	0.03377 0.00266	0.02671 0.00517	0.000^*^	0.03249 0.00237	0.02497 0.00398	0.000^*^
Thalamus total, left	0.32430 0.02209	0.32525 0.02228	0.882	0.33248 0.01846	0.32428 0.02484	0.433	0.32998 0.02614	0.31843 0.02588	0.431
LGN	0.00705 0.00084	0.00701 0.00066	0.894	0.00727 0.00159	0.00688 0.00119	0.475	0.00713 0.00085	0.00658 0.00115	0.220
MGN	0.01009 0.00150	0.10103 0.00082	0.898	0.01017 0.00133	0.00965 0.00126	0.320	0.01081 0.00133	0.00981 0.00114	0.106
Anterior nuclei	0.00548 0.00092	0.00539. 00086	0.665	0.00531 0.00057	0.00555 0.00087	0.328	0.00533 0.00072	0.00575 0.00100	0.257
Central nuclei	0.01578 0.00014	0.01538 0.00151	0.447	0.01597 0.00137	0.01500 0.00143	0.158	0.01624 0.00188	0.01491 0.00165	0.177
Lateral dorsal nuclei	0.00276 0.00073	0.00295 0.00080	0.418	0.00283 0.00074	0.00324 0.00054	0.086	0.00307 0.00072	0.00332 0.00069	0.252
Lateral posterior nuclei	0.02229 0.00299	0.02363 0.00275	0.128	0.02276 0.00238	0.02432 0.00167	0.108	0.02363 0.00197	0.02401 0.00233	0.695
Medial dorsal nuclei	0.05222 0.00562	0.05085 0.00544	0.415	0.05292 0.00427	0.05046 0.00626	0.336	0.05276 0.00650	0.04920 0.00605	0.290
Pulvinar	0.07683 0.00768	0.07732 0.00654	0.798	0.07540 0.00570	0.07350 0.00848	0.565	0.07474 0.00625	0.07162 0.00742	0.556
VA	0.03156 0.00297	0.03175 0.00404	0.868	0.03288 0.00229	0.03343 0.00302	0.550	0.03199 0.00340	0.03234 0.00350	0.979
VP	0.02520 0.00265	0.02484 0.00230	0.631	0.02593 0.00264	0.02329 0.00244	0.015	0.02547 0.00208	0.02202 0.00264	0.009
VL	0.04554 0.00444	0.04591 0.00551	0.810	0.04858 0.00363	0.04578 0.00585	0.156	0.04691 0.00351	0.04434 0.00674	0.268
Thalamus total, right	0.32413 0.02336	0.32019 0.02076	0.567	0.33124 0.01278	0.31827 0.02027	0.083	0.32838 0.02470	0.31547 0.02189	0.257
LGN	0.01136 0.00138	0.01143 0.00134	0.861	0.01190 0.00125	0.01203 0.002108	0.947	0.01177 0.00096	0.01160 0.00211	0.626
MGN	0.01235 0.00142	0.01226 0.00109	0.790	0.01268 0.00096	0.01262 0.00148	0.875	0.01314 0.00120	0.01264 0.00181	0.581
Anterior nuclei	0.00759 0.00109	0.00720 0.00146	0.283	0.00749 0.00070	0.00792 0.00068	0.209	0.00787 0.00099	0.00796 0.00055	0.912
Central nuclei	0.00979 0.00084	0.00948 0.00078	0.218	0.01003 0.00060	0.00968 0.00075	0.237	0.01021 0.00117	0.00960 0.00073	0.224
Lateral dorsal nuclei	0.00309 0.00089	0.00296 0.00077	0.570	0.00299 0.00058	0.00329 0.00064	0.180	0.00302 0.00063	0.00334 0.00060	0.084
Lateral posterior nuclei	0.01683 0.00247	0.01672 0.00216	0.886	0.01594 0.00158	0.01687 0.00132	0.213	0.01708 0.00147	0.01680 0.00124	0.988
Medial dorsal nuclei	0.04785 0.00373	0.04630 0.00452	0.226	0.04823 0.00430	0.04647 0.00435	0.370	0.04928 0.00685	0.04610 0.00474	0.222
Pulvinar	0.09388 0.00827	0.09504 0.00743	0.569	0.09443 0.00553	0.08914 0.00799	0.114	0.09228 0.00659	0.08769 0.00847	0.307
VA	0.03362 0.00285	0.03242 0.00400	0.246	0.03532 0.00326	0.03405 0.00280	0.261	0.03404 0.00323	0.03352 0.00297	0.509
VP	0.03795 0.00441	0.02331 0.00260	0.753	0.02382 0.00180	0.02196 0.00257	0.047	0.02337 0.00163	0.02135 0.00253	0.054
VL	0.03876 0.00349	0.03715 0.00435	0.221	0.04071 0.00302	0.03818 0.00468	0.109	0.03925 0.00388	0.03786 0.00485	0.339
Striatum total, left	0.51914 0.03450	0.49659 0.02327	0.013^*^	0.51636 0.03540	0.50261 0.03202	0.482	0.49783 0.02986	0.50050 0.03693	0.936
Striatum total, right	0.51211 0.03543	0.48445 0.02920	0.006^*^	0.50521 0.03297	0.49679 0.02506	0.557	0.49142 0.02774	0.49558 0.02767	0.917

Values represent means and standard deviations of volumes relative to estimated total incranial volume ^*^100. HC, healthy control subjects; MCI-S, stable mild cognitive impairment at baseline; MCI-CB, mild cognitive impairment converters at baseline; MCI-CC, mild cognitive impairment converters at time of conversion; eTIV, estimated total intracranial volume; CA, cornu ammonis; LGN, lateral geniculate nucleus; MGN, medial geniculate nucleus; VA, ventral anterior nuclei; VP, ventral posterior nuclei; VL, ventral lateral nuclei.

* *Significant p value test by using volumes relative to estimated total intracranial volume and multiplied by 100 as dependent variables, and including age and gender in the model, and after correction for multiple testing*.

**Table 3:** **Raw volume sizes for patient and control groups**.

	**Group 1**		***p***	**Group 2**		***p***	**Group 3**		***p***
	**HC**	**MCI-S**		**HC**	**MCI-CB**		**HC**	**MCI-CC**	
eTIV, cm^3^	1586.33 177.35	1546.71 155.10	0.424	1499.99 155.00	1498.05 133.58	0.976	1487.72 132.59	1507.66 133.52	0.741
Hippocampus total, left	2238.28 260.63	2150.25 434.33	0.390	2250.76 374.17	1687.81 274.65	0.003^*^	2156.61 223.12	1609.17 247.38	0.000^*^
Subiculum	332.02 44.55	304.09 62.10	0.052	331.47 51.73	248.01 43.84	0.001^*^	305.25 29.47	238.94 48.22	0.002^*^
CA1	716.61 83.99	706.75 141.74	0.785	727.14 132.45	548.12 89.81	0.004^*^	692.32 81.86	542.17 95.77	0.004^*^
CA2-CA3	133.94 27.55	130.89 31.18	0.729	132.45 31.93	111.93 21.82	0.150	134.26 16.94	98.11 20.69	0.001^*^
CA4/Dentate gyrus	534.68 64.87	503.78 101.15	0.215	538.14 86.87	414.63 75.53	0.006^*^	522.28 73.58	390.57 67.95	0.001^*^
Strata	521.03 69.33	504.74 118.25	0.564	521.85 93.31	365.12 67.27	0.001^*^	502.50 56.59	339.38 50.29	0.000^*^
Hippocampus total, right	2234.65 300.09	2232.02 340.64	0.993	2244.32 274.42	1858.63 335.92	0.019^*^	2149.88 196.89	1775.68 280.26	0.006^*^
Subiculum	304.93 34.75	293.59 52.12	0.377	311.29 45.88	251.38 54.78	0.027	296.18 38.27	239.80 47.82	0.009^*^
CA1	743.04 108.48	751.13 117.96	0.782	750.52 107.06	638.02 115.92	0.057	722.65 74.27	628.90 115.45	0.073
CA2-CA3	146.56 23.46	147.91 27.64	0.844	143.55 22.07	125.88 26.26	0.119	138.63 29.32	111.37 26.32	0.057
CA4/Dentate gyrus	536.26 73.05	534.87 76.72	0.980	531.92 70.60	443.88 73.45	0.017	509.88 61.47	420.12 57.92	0.008^*^
Strata	503.86 85.79	504.52 95.45	0.945	507.04 57.00	399.47 85.29	0.007^*^	482.54 45.84	375.49 64.85	0.001^*^
Thalamus total, left	5126.73 503.34	5036.59 652.23	0.565	4977.11 470.58	4854.86 547.58	0.886	4887.31 330.22	4792.43 195.68	0.870
LGN	111.36 15.48	108.48 15.57	0.522	108.08 19.89	102.21 14.05	0.525	105.67 13.19	98.15 11.26	0.258
MGN	159.11 24.28	156.87 22.00	0.706	151.80 18.91	143.73 15.72	0.404	160.02 17.57	146.96 12.99	0.148
Anterior nuclei	87.56 19.90	83.39 15.61	0.361	79.87 13.58	83.20 15.85	0.241	78.88 9.53	86.78 18.23	0.104
Central nuclei	248.49 27.52	237.58 31.05	0.147	239.37 30.15	224.34 25.28	0.335	240.92 30.16	223.86 23.09	0.871
Lateral dorsal nuclei	44.53 14.55	45.86 13.94	0.761	42.85 13.33	48.56 9.44	0.083	45.40 10.29	50.02 11.52	0.105
Lateral posterior nuclei	355.06 68.93	366.43 60.36	0.542	341.19 47.86	364.56 43.91	0.099	349.79 21.34	361.28 42.25	0.331
Medial dorsal nuclei	822.66 79.53	785.62 110.11	0.178	790.30 67.28	753.79 102.32	0.501	779.15 65.12	738.24 83.74	0.508
Pulvinar	1212.08 126.63	1195.09 146.50	0.655	1128.08 113.66	1101.08 160.98	0.940	1109.93 118.10	1079.69 146.41	0.800
VA	500.06 67.78	493.17 89.54	0.755	493.21 61.80	500.08 56.84	0.367	472.95 33.09	485.61 49.05	0.399
VP	396.65 33.99	383.97 50.83	0.275	386.85 34.62	348.36 44.33	0.050	377.78 34.68	331.26 44.02	0.041
VL	719.09 80.65	711.71 117.47	0.799	725.58 56.69	684.07 92.85	0.283	695.61 56.70	665.58 93.58	0.551
Thalamus total, right	5122.67 493.57	4958.83 643.05	0.280	4959.92 448.02	4758.09 417.01	0.423	4864.32 321.10	4742.79 377.55	0.839
LGN	179.37 23.03	176.95 28.31	0.763	177.46 14.77	178.81 25.88	0.873	174.31 10.92	173.45 23.64	0.928
MGN	195.04 25.38	190.02 29.13	0.439	189.86 19.72	188.17 20.73	0.882	195.39 25.37	189.52 24.60	0.956
Anterior nuclei	120.72 22.93	111.68 26.22	0.168	112.40 16.29	119.18 18.36	0.276	116.68 15.55	120.22 15.19	0.468
Central nuclei	154.73 17.33	146.89 21.11	0.118	150.24 16.51	144.89 16.56	0.648	151.41 18.34	144.54 14.79	0.625
Lateral dorsal nuclei	49.43 15.49	45.96 13.21	0.374	45.14 11.29	49.85 12.83	0.196	45.27 11.65	50.67 11.98	0.087
Lateral posterior nuclei	267.29 47.20	259.55 47.31	0.571	239.24 34.40	252.46 28.41	0.234	253.76 28.45	252.63 23.74	0.189
Medial dorsal nuclei	756.02 74.65	716.16 99.86	0.104	720.75 68.41	693.40 61.79	0.503	727.41 73.61	691.37 54.93	0.385
Pulvinar	1484.26 165.02	1469.57 183.34	0.749	1414.45 145.15	1331.68 137.33	0.227	1370.91 138.16	1316.74 122.95	0.781
VA	532.09 65.62	503.16 89.00	0.204	527.86 53.61	510.49 66.36	0.670	503.71 35.07	504.70 56.99	0.813
VP	366.80 32.69	357.21 50.58	0.402	356.18 34.07	327.49 34.56	0.069	346.68 29.03	320.19 32.51	0.123
VL	611.33 14.59	576.04 95.90	0.156	608.29 51.25	569.12 60.62	0.176	581.08 52.21	567.77 62.64	0.684
Striatum total, left	8208.75 818.54	7688.01 897.51	0.031^*^	7746.97 1003.05	7513.04 651.33	0.844	7389.66 615.77	7527.29 689.44	0.356
Striatum total, right	8095.68 805.24	7497.72 905.81	0.015^*^	7575.03 917.71	7432.17 645.25	0.989	7303.52 696.77	7453.16 554.69	0.382

*Values represent means and standard deviations of raw volumes mm3. HC, healthy control subjects; MCI-S, stable mild cognitive impairment at baseline; MCI-CB, mild cognitive impairment converters at baseline; MCI-CC, mild cognitive impairment converters at time of conversion; eTIV, estimated total intracranial volume; CA, cornu ammonis; LGN, lateral geniculate nucleus; MGN, medial geniculate nucleus; VA, ventral anterior nuclei; VP, ventral posterior nuclei; VL, ventral lateral nuclei*.

* *Significant p value test by using raw volumes as dependent variable, and including age and gender in the model, and after correction for multiple testing*.

### Volumetric analyses in MCI-CB

In contrast, pronounced reductions in volumes relative to eTIV were found in MCI-CB (see Table 2). In particular, and apart from CA2-CA3 volumes, all bilateral hippocampal subfield volumes were smaller in MCI-CB compared to HC (right CA1 *t* = 3.23, *p* = 0.005; subiculum *t* = 3.29, *p* = 0.005; CA4/Dentate gyrus *t* = 3.80, *p* = 0.002; strata *t* = 4.55, *p* < 0.001/left CA1 *t* = 5.18, *p* < 0.001; subiculum *t* = 4.96, *p* < 0.001; CA4/Dentate gyrus *t* = 4.66, *p* < 0.001; strata *t* = 5.91, *p* < 0.001; *df* = 3,16) after correction for multiple testing. With regard to thalamic subnuclei, and although statistically significant, effects of volume reductions in bilateral VP in MCI-CB (right *p* = 0.047, left *p* = 0.015) did not survive the correction for multiple testing. Significance and *p*-values were similar when using raw volumes instead of volumes relative to eTIV. However, some of the right hemispheric differences in hippocampal subfield volumes did not quite achieve the level of significance (CA1 *p* = 0.057). Additionally, and although statistically significant, two right hemispheric effects did not survive the correction for multiple comparisons (CA4/Dentate gyrus *p* = 0.017; subiculum *p* = 0.027) (see Table 3).

### Volumetric analyses in MCI-CC

Further extended reductions in volumes relative to eTIV revealed in MCI-CC (see Table 2). More precisely, and after correction for multiple testing, all bilateral hippocampal subfield volumes were smaller in MCI-CC compared to HC (left CA1 *t* = 5.09, *p* < 0.001; subiculum *t* = 5.00, *p* < 0.001; CA2-CA3 *t* = 4.01, *p* = 0.001; CA4/Dentate gyrus *t* = 6.66, *p* < 0.001; strata *t* = 9.41, *p* < 0.001/right CA1 *t* = 3.24, *p* = 0.005; subiculum *t* = 3.91, *p* = 0.001; CA2-CA3 *t* = 2.18, *p* = 0.044; CA4/Dentate gyrus *t* = 4.47, *p* < 0.001; strata *t* = 5.53, *p* = 0.000; *df* = 3,16). Similar as in MCI-CB, effects of VP volume reductions in MCI-CC (right *p* = 0.054, left *p* = 0.009) did not survive the correction for multiple testing. Significance and *p*-values were similar when using raw volumes instead of volumes relative to eTIV. However, two right hemispheric effects in hippocampal subfield volumes did not quite achieve the level of significance (CA1 *p* = 0.074; CA2-CA3 *p* = 0.057) (see Table 3).

### Vertex-wise cortical thickness analyses in MCI groups

**Figure 3:**
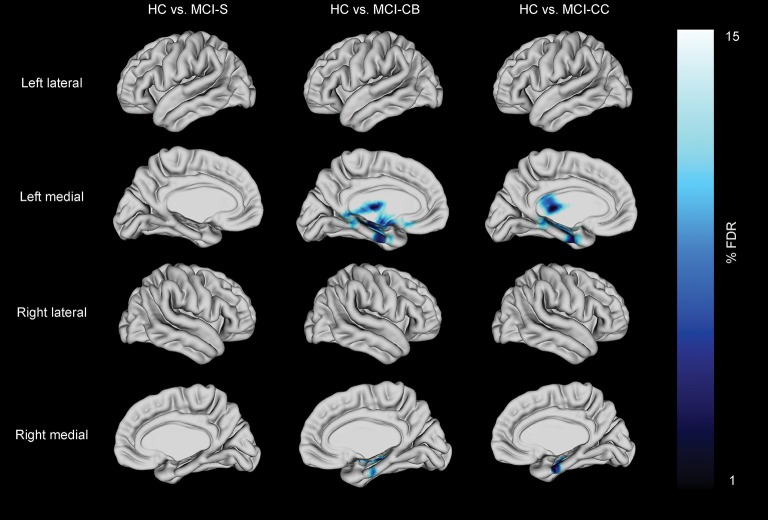
**Cortical thickness differences in patients with stable mild cognitive impairment (MCI-S), future converters at baseline (MCI-CB) and converters at time of conversion (MCI-CC) when compared with healthy control subjects (HC)**. Images were generated after including age and gender in the model, and after correction using FDR at *q* = 0.15 to better illustrate the anatomical localization. Bar shows FDR-values, with blue /light blue indicating reduced cortical thickness.

**Figure 4:**
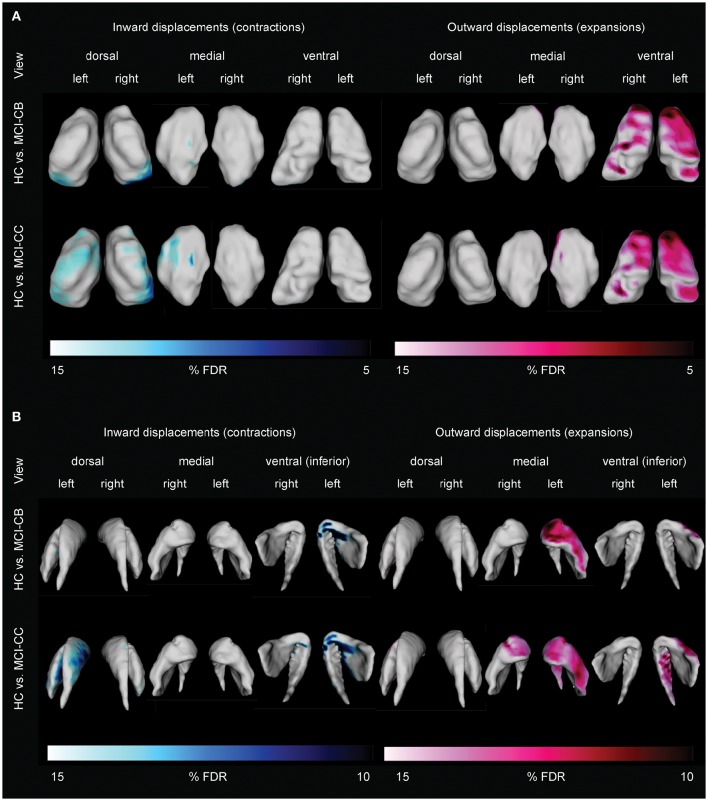
**Differences in thalamic (A)** and striatal **(B)** shape alterations in future converters at baseline (MCI-CB) and at time of conversion (MCI-CC) when compared with healthy control subjects (HC). Images were generated after including age and gender in the model, and after correction using FDR at *q* = 0.15 to better illustrate the anatomical localization. Bars show FDR-values, with blue/light blue indicating inward displacements (contractions) and pink/light pink indicating outward displacements (expansions).

Analyses on data corrected for multiple testing by using FDR at *q* < 0.10 revealed no cortical thinning in MCI-S compared to HC. Reduced cortical thickness, however, was found in MCI-CB and MCI-CC compared to HC (Figure 3, Table 4). Significant effects were limited to medial areas such as the left parahippocampal cortex, left subgenual cingulate, and left region of the uncus in MCI-CB. Similar regions revealed cortical thinning in the right hemisphere, with significance only at *q* < 0.15 though (Figure 4). Importantly, the pattern of cortical thinning extended to the right hemisphere in MCI-CC, where cortical thinning in bilateral parahippocampal cortices and bilateral regions of the uncus now achieved an appropriate level of significance.

**Table 4:** **Reduced cortical thickness in patient groups**.

**Anatomic localization**	**MNI coordinates (peak)**			***t* statistic (peak)**
	**x**	**y**	**z**	
**A**				
Parahippocampal Cortex, left	−30	−16	−27	−5.77^**^
Subgenual Cingulate, left	−18	15	−14	−4.34^**^
Uncus, left	−29	−29	−8	−6.53^**^
**B**				
Parahippocampal Cortex, left	−25	−13	−32	−7.13^**^
Uncus, left	−31	−12	−23	−7.06^**^
Parahippocampal Cortex, right	29	−12	−28	−5.45^*^
Uncus, right	31	−10	−22	−5.20^*^

*Anatomical localization of cortical thinning in mild cognitive impairment converters at baseline **(A)**, and in mild cognitive impairment converters at time of conversion **(B)**. MNI: Montreal Neurological Institute*.

* *significant after including age and gender in the model, and after correction using False Discovery Rate at q < 0.10*.

** *significant after including age and gender in the model, and after correction using False Discovery Rate at q < 0.01*.

### Vertex-wise subcortical shape analyses in MCI groups

Analyses on data corrected for multiple testing by using FDR at *q* < 0.10 revealed no striatal or thalamic shape alterations in MCI-S, but pronounced striatal and thalamic displacements in MCI-CB and MCI-CC compared to HC.

Thalamic contractions and expansions are presented in Figure 4A. In contrast to MCI-S, MCI-CB, and MCI-CC revealed contractions which were limited to dorsal and medial parts, and were more pronounced in the left than in the right hemisphere. Specifically, MCI-CB exhibited contractions in bilateral dorsal aspects of the pulvinar, bilateral dorsal aspects of VP, and in left medial aspects of VP and medial dorsal nuclei. Again, the pattern of alterations had further continued in MCI-CC, exhibiting more pronounced contractions extending from dorsal aspects of the pulvinar and VP to dorsal aspects of VL, lateral posterior nuclei, and VA in the right hemisphere. In contrast, contractions in the left hemisphere were now limited to dorsal aspects of VL, VA, and medial dorsal nuclei. However, there was a tendency toward significant contractions (*q* = 0.15) in dorsal aspects of the pulvinar, VP and lateral posterior nuclei as well. Thalamic expansions in turn were limited to ventral and medial parts. MCI-CB revealed expansions in bilateral ventral aspects of the central nuclei, VA, VL, and VP. MCI-CC revealed the same, though more pronounced pattern of expansions in both hemispheres, with additional expansion in the medial aspect of the medial dorsal nuclei.

Striatal displacements are presented in Figure 4B. Again in contrast to MCI-S, the other groups displayed contractions, predominantly in the left hemisphere and most pronounced in ventral (inferior) aspects. More precisely, MCI-CB revealed contractions in medial parts of the putamen and anterior parts of the striatum (caudate head). The same pattern was found in MCI-CC, with more pronounced alterations in left ventral (inferior) medial parts of the putamen and with continued spreading to the left dorsal medial striatum (caudate body) and to right ventral (inferior) aspects of the anterior striatum (caudate head). Similar to the contractions, striatal expansions were more pronounced in the left hemisphere: MCI-CB showed pronounced expansions in ventral aspects of the anterior striatum (caudate head) and lateral putamen. MCI-CC showed a similar pattern, but with further continued expansions to ventral (inferior) aspects of the left striatum (caudate tail), and to ventral aspects of the anterior striatum (caudate head) of the right hemisphere.

### Discriminative accuracy of shape alterations and hippocampus volumes

**Figure 5:**
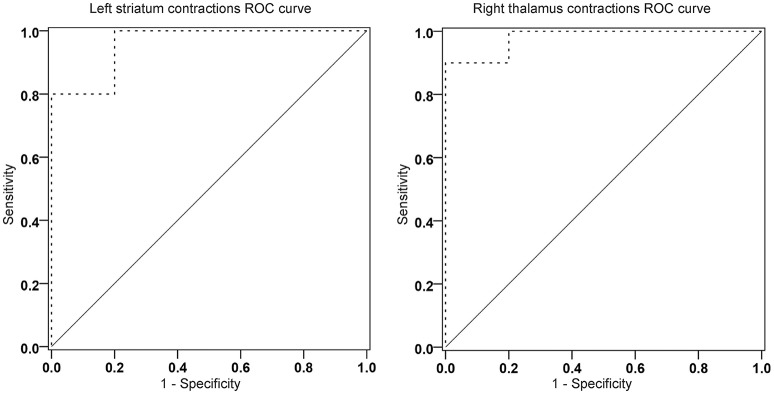
**Receiver operating characteristic curve analyses of contractions in the left striatum (area under the curve [AUC] = 0.96, ***p*** < 0.001) and the right thalamus (AUC = 0.98, ***p*** < 0.001) for discriminating future converters at baseline from healthy control subjects**. ————- ROC curvereference lines.

For these analyses, between group t statistics from comparison between HC and MCI-CB were used. More precisely, for all vertices whose *t*-values constituted a local minimum (or maximum), the average of the displacement values in mm was computed for each subject. This was done separately for each structure and hemisphere. The analyses revealed contractions of the right thalamus and left striatum having an AUC of 0.98 (95% confidence interval [CI] 0.929-1.000, *p* < 0.001) and of 0.96 (95%CI 0.883-1.000, *p* < 0.001) for discriminating HC from MCI-CB (Figure 5), both AUC confirming excellent accuracy and high statistical significance similar to left hippocampus volume (raw volume AUC = 0.95, 95%CI 0.861-1.000, *p* < 0.001; volume relative to eTIV AUC = 0.95, 95%CI 0.848-1.000, *p* < 0.001) and increased accuracy and statistical significance when compared to the right hippocampus volume (raw volume AUC = 0.80, 95%CI 0.572-1.000, *p* < 0.05; volume relative to eTIV AUC = 0.84, 95%CI 0.633-1.000, *p* < 0.05). The same AUC analyses carried out for thalamic expansions (left AUC = 0.92 95%CI 0.767-1.000, *p* = 0.001; right AUC = 0.93 95%CI 0.816-1.000, *p* = 0.001), thalamic contractions left (AUC = 0.86, 95%CI 0.685-1.000, *p* = 0.007) and striatal expansions left (AUC = 0.88, 95%CI 0.730-1.000, *p* = 0.004) revealed lower but still statistically significant accuracy for discriminating HC from MCI-CB.

## Discussion

In the current study, we used a combination of novel (thalamic and striatal shape indices) and well-established (cortical thickness and the volume of the hippocampus and its subfields) structural imaging techniques to characterize the neuroanatomy of MCI-to-AD converters. We examined the neuroanatomy in the MCI-to-AD converters using data acquired both at baseline and time of conversion. In addition, we compared baseline data from stable MCI subjects, comparing them with data from group-wise matched HC in a cross-sectional manner.

### Cortical thinning in future converters

There was no significant difference in cortical thickness in MCI-S compared to HC, but in MCI-CB in left parahippocampal regions including the uncus with further propagation to bilateral regions in MCI-CC. This pattern is consistent with the literature (Braak and Braak, 1991b; Mitchell et al., 2002) and with morphometric changes previously associated with early stages of AD (Mitchell et al., 2002; Drago et al., 2011). Like others (Lebedev et al., 2013), we also identified cortical thinning in the subgenual cingulate region in MCI-CB. Functionally, this region has been related to normal sadness reactions (Phan et al., 2002), and reduced volumes have been observed in patients with major depressive disorder (Drevets et al., 2008). Although clinically significant depression was one of the exclusion criteria in our study, very subtle and subclinical depressive symptoms that are difficult to quantify may be related to this finding in MCI-CB, leaving the exact mechanism unclear. This region has furthermore been associated with the uptake of serotonin (Lanzenberger et al., 2013). Serotonin is involved in the regulation of sleep (Portas et al., 2000) which is disturbed in AD (Westerberg et al., 2012).

### Hippocampal atrophy in future converters

MCI-S revealed no global or local hippocampal volume reductions compared to HC. However, in accordance with the AD literature (Stepan-Buksakowska et al., 2014) and our expectations, we found reduced volumes of bilateral hippocampi in MCI-CB and MCI-CC as well as reduced local volumes of all but one (CA2-CA3) bilateral subfields already in MCI-CB, and of all bilateral subfields in MCI-CC.

Hippocampal atrophy represents the key imaging marker in AD research. The successful identification of hippocampal subfields by using high-field MRI, however, has offered a more refined approach (Mueller and Weiner, 2009; Yushkevich et al., 2009; Mueller et al., 2010; Antharam et al., 2012; Wisse et al., 2014a). Advances in segmentation and analysis techniques have now enabled the field to identify hippocampal subfield alterations on images obtained from standard clinical systems. Corresponding with the pattern of neurofibrillary tangle formation, predominant CA1 atrophy has been found in MCI (Apostolova et al., 2006a; Atienza et al., 2011; Pluta et al., 2012; La Joie et al., 2013; Yushkevich et al., 2015a), with some of these studies having reported additional subicular alterations (Atienza et al., 2011; La Joie et al., 2013) and alterations in additional subfields (Amaral et al., 2016). A more extended pattern also including CA2 and CA3 or even CA4/Dentate gyrus has been identified in AD (Apostolova et al., 2006a; Frisoni et al., 2008; Frankó et al., 2013; Li et al., 2013). Importantly, alterations in these subfields have been related to impaired memory functions in amnestic MCI (Atienza et al., 2011; Ferrarini et al., 2014). Local analysis of the structure, therefore, has been suggested advantageous for the early detection of dementia (Tang et al., 2014).

Nevertheless, only a few studies have examined hippocampal subfields in future converters. Even though they have applied surface-based subfield imaging without providing volumetric information, results of alterations in CA1 and/or subiculum in future converters to MCI (Apostolova et al., 2010) or to AD (Apostolova et al., 2006b; Chételat et al., 2008; Frankó et al., 2013) are in agreement with our own findings. In line with further progression of tangle accumulation (Braak and Braak, 1991b), we found similar bilateral CA2-CA3 volumes in MCI-CB but reduced bilateral CA2-CA3 volumes in MCI-CC compared to HC. Additionally, we found bilateral CA4/Dentate gyrus volumes were lowered not only in MCI-CC but also in MCI-CB. Although CA4/Dentate gyrus volume reduction has mainly been identified in dementia stages (Frankó et al., 2013; Li et al., 2013), earlier alterations in this subfield have also been reported by others (Pluta et al., 2012). We also identified reduced volumes of bilateral strata lacunosum/radiatum/moleculare in MCI-CB and MCI-CC, and our results are supported by studies reporting stratum radiatum and stratum lacunosum/moleculare of CA1 being affected by early tangle accumulation (Braak and Braak, 1997; Thal et al., 2000; Braak et al., 2006) and atrophy in mild AD (Kerchner et al., 2010). It shoud be further noted that only a few protocols exist allowing for the reliable, automated segmentation of hippocampal subfields (see Wisse et al., 2014b for a critical discussion; Yushkevic et al., 2015b). Here, we used a multi-atlas based segmentation approach (Chakravarty et al., 2013; Voineskos et al., 2015) that performs as well as other methods used in the field (Pipitone et al., 2014).

### Thalamic alterations in future converters

MCI-S did not reveal any deviation from HC, neither in thalamic total or subfield volumes nor in shape alterations. Contrary to our expectation, the same was true for both total and local thalamus volumes in MCI-CB and MCI-CC. Indeed reduced thalamic total volumes have been reported in amnestic MCI (Hahn et al., 2016) and MCI-CB (Bossa et al., 2011). However, thalamic atrophy in AD is debated, particularly in early disease stages (Xuereb et al., 1991; Braak and Braak, 1991a; Paskavitz et al., 1995), where only a few regions are affected by a small number of isolated neurofibrillary tangles (Braak and Braak, 1991b). Moreover, our results are in agreement with other studies having observed similar volumes in amnestic MCI, MCI and AD patients (Cho et al., 2014).

But first and foremost, and despite the absence of volumetric differences, we identified a widespread pattern of shape alterations in MCI-CB and MCI-CC with displacements showing similar if not improved discrimination between HC and MCI-CB compared to hippocampus volume. These findings may represent a critical MRI-based marker for AD. Confirming our expectation, the pronounced thalamic shape alterations in VA found in MCI-CB and MCI-CC cover regions that are affected from early neurofibrillary tangles and later occurring amyloid deposition (Braak and Braak, 1991b). Importantly, the thalamus plays a crucial role in the Papez circuit with the anterior thalamus and the pulvinar, both having shown shape alterations in MCI-CB and MCI-CC, being directly connected to the hippocampus (Zarei et al., 2010). Furthermore, frontostriatal circuits link dorsolateral prefrontal, anterior cingulate, and orbitofrontal cortex regions (Alexander et al., 1986) via the striatum / globus pallidus (Haber, 2003) to VA and medial dorsal aspects of the thalamus (Tekin and Cummings, 2002; Bonelli and Cummings, 2007), aspects that again have shown shape alterations in MCI-CB and MCI-CC. Further significant shape alterations were found in the VP, VL and lateral posterior nuclei connecting the structure with the somatosensory, motor, premotor and prefrontal and temporal and parietal cortices (Behrens et al., 2003; De bourbon-Teles et al., 2014). Consistent with these extensive connections, thalamic regions affected from shape alterations in MCI-CB and MCI-CC have been linked with memory and frontal executive, attention, visuospatial perception, and emotion processing (Wilke et al., 2013; Saalmann, 2014; De bourbon-Teles et al., 2014; Arend et al., 2015), with all of these functions being impaired early in AD (Klekociuk et al., 2014).

To our knowledge, there has been only one other study investigating subcortical shape alterations in MCI-S and MCI-CB, without a HC group (Tang et al., 2014). The contractions found in the pulvinar and dorsal aspects of the VP in MCI-CB are consistent with contractions that have been identified in amnestic MCI by an earlier study of our group (Leh et al., 2015). Additionally, the authors have documented contractions in regions that remained unaffected in the present MCI-CB sample such as the VL and lateral posterior nuclei. These, however, revealed contractions in the MCI-CC group. Our finding of contractions in dorsal, medial dorsal and anterior regions with more pronounced results in the left than in the right hemisphere in MCI-CB and MCI-CC, contractions in the pulvinar and dorsal as well as medial dorsal regions in MCI-CC in turn are consistent with other study results (Qiu et al., 2009; Cho et al., 2014; Stepan-Buksakowska et al., 2014; Hahn et al., 2016). The few results about thalamic expansions in MCI and AD are inconsistent. In contrast to expansions in ventral regions in both MCI-CB and MCI-CC, the earlier study from our group has identified expansions in more lateral aspects in amnestic MCI, whereas others have reported expansions in medial or even dorsal aspects in MCI and AD (Qiu et al., 2009). Further studies are warranted to find out whether there is a typical AD-related pattern of thalamic expansion.

### Striatal alterations in future converters

Against our expectation, we observed reduced bilateral striatal volumes in MCI-S but similar volumes in MCI-CB and MCI-CC when compared to HC. The most plausible explanation for this seem to be age- and gender-related shrinking, or age-related changes in dopamine and frontostriatal networks (Klostermann et al., 2012) resulting in reduced striatal volumes. Due to age- and gender-matched MCI-S and HC groups, however, this is rather unlikely in the present study. Rather, the result may indicate non-AD pathology in our MCI-S sample. This assumption is further supported by cognitive profiles differing between MCI-S and MCI-CB. In comparison with their respective HC group, MCI-S showed significantly reduced cognitive scores not only in memory tests but also in tests assessing executive functions, attention and language abilities (see Table 1). In contrast, and apart from impaired memory functions, MCI-CB performed worse than their respective HC in the category fluency task and in the boston naming test but not in attentional tasks. Both, the category fluency task and the boston naming test have closely been linked with semantic knowledge (Delis and Kaplan, 2001), which in turn is thought to rely on temporal brain regions (Levy et al., 2004) known to be affected early in AD (Tondelli et al., 2012). Genereally speaking, MCI-S in our study might indeed represent non-AD cognitive impairment mainly based on attentional deficits influencing other cognitive processes and leading to a more widespread pattern of cognitive impairment whereas our MCI-CB group showed an AD-typical pattern of cognitive impairment.

Normal striatal volumes in MCI-CB and MCI-CC in turn are most likely attributable to the early disease stages of each group: striatal amyloid deposition and tangle formation have evidenced at late histopathological disease stages, and mainly after dementia onset (Braak and Braak, 1990, 1991a,b; Thal et al., 2002; Beach et al., 2012). Hence, the striatum in MCI-CB and MCI-CC may still be free of typical AD pathology. Correspondingly, similar volumes of the striatum, putamen and caudate in MCI and future converters compared to HC have been reported by others (Bossa et al., 2011; Roh et al., 2011).

As expected, however, shape analyses showed no alterations in the striatum in MCI-S but pronounced displacements in MCI-CB and MCI-CC. The observed shape alterations were limited to the left striatum in MCI-CB, but displacements had propagated within the structure and to the right hemisphere in MCI-CC. Furthermore, and as with the thalamic shape alterations, these striatal shape alterations revealed similar abilities for discriminating HC from MCI-CB compared to hippocampus volume. Aspects of the striatum showing most pronounced contractions and expansions such as the caudate head, body and tail as well as medial and lateral putamen have been linked to a wide range of cognitive functions. These regions are involved in attention, planning and memory (Cummings, 1995), all of which are impaired early in AD (Klekociuk et al., 2014).

Again, the study from Tang et al. (2014) represents the only study we are aware of having documented shape alterations in MCI-S and MCI-CB; however no HCs were compared in this study. In agreement with other studies, however, they demonstrated patterns of striatal contractions in MCI and AD patients (Qiu et al., 2009; Tang et al., 2014; De Jong et al., 2014) that were comparable to the patterns found in the current study. Interestingly, only a few studies have documented striatal expansions in general (Tang et al., 2014; De Jong et al., 2014), though these expansions were less pronounced than in the present study (visual inspection).

It is beyond the aim of our study to draw direct inferences about the neuronal correlate of shape alterations. Given the pronounced connections with other disease-relevant structures (Leh et al., 2008), however, striatal and thalamic shape alterations may represent secondary downstream effects. A similar effect has been proposed by Stepan-Buksakowska et al. (2014). Specifically, volume reductions in the hippocampus and other early affected cortical regions may have caused subsequent morphometric changes in connected regions such as the thalamus and the striatum, without generating volume reductions yet. Although we cannot rule out the possibility of contractions representing atrophy-related alterations, the applied surface-based shape analyses provide local, but not fully comprehensive information about the entire structure. Accordingly, and as it has been shown in the present study, structural shape alterations are not necessarily associated with corresponding volume changes. Hence, our results of shape differences in the absence of volume differences highlight the importance of considering shape changes along with established volume measures.

## Limitations

The authors are fully aware of other studies investigating subcortical shape alterations in MCI and/or AD patients (Cho et al., 2014; Stepan-Buksakowska et al., 2014; Tang et al., 2014; Hahn et al., 2016). However, and apart from the study from Tang et al. (2014), we are not aware of other studies investigating thalamic and striatal shape abnormalities in MCI-S, MCI-CB, and MCI-CC in a single comparative study, and of no study comparing these shape abnormalities with measures from age—and gender matched HC. More importantly, we are not aware of any other study providing evidence of thalamic/striatal shape alterations at such early stages of AD (MCI-CB)—with absent thalamic/striatal shape alterations in MCI-S at the same time. Thus, our work provides support for these novel morphometric measures representing a potential and very sensitive early marker for AD. Additionally, and compared with emerging techniques such as hippocampal subfield volume analysis and already established techniques such as cortical thickness analysis, shape analysis represents an advanced and novel technique. Accordingly, the number of studies investigating subcortical shape alterations in MCI/AD subjects is limited (i.e., Qiu et al., 2009; Cho et al., 2014; De Jong et al., 2014; Tang et al., 2014; Hahn et al., 2016). Furthermore, the comparability of results remains difficult due to different imaging processing algorithms and different MRI acquisition modalities (i.e., with field strength of scanners varying from 1.5 T in our own study to 7 T in the study from Tang et al., 2014, and with other high-sample studies using images acquired from various scanners in the study from Qiu et al., 2009). Thus, contractions and expansions represent a novel way to quantify the morphometry of brain regions, with shape alterations having been associated with memory performance in our previous study (Voineskos et al., 2015).

Although other groups have also reported shape analysis results obtained from studies consisting of similarly small sample sizes (i.e., Stepan-Buksakowska et al., 2014, 12 AD, 13 HC), the small group sizes may have prevented the detection of small effects in this study. However, we believe that the reliability of our findings are supported by low standard deviations across our measures (see Tables 2, 3) as well as by the overall characteristic of the results obtained using well-established analyses techniques (the volume of the hippocampus and its subfields and cortical thickness) and being perfectly in line with the literature. Furthermore, statistically significant differences in shape alterations between small groups indicate that even more pronounced effects might be expected in future studies using larger sample sizes. In sum, and despite the small group sizes, we consider the present study results important to investigate whether shape alteration can help localizing differences associated with the stability or progression of cognitive impairment. However, further studies are required to confirm our results in larger samples. This is also true for our ROC analyses, whose AUC values were remarkably high for measures of striatal and thalamic shape and left hippocampus and may represent an overestimation of discriminative ability due to small sample size.

The approach of using three different control groups might indeed increase chances of coincidental findings, and thus represent a limitating factor. However, and for the present study, MCI subjects were selected from a study aiming to characterize future converters to AD. The study did not consist of HC. Therefore, it was necessary to recruit HC restrospectively, rendering the acquisition of longitudinal data and performance of longitudinal statistical analyses impossible. Furthermore, MCI-CB differed from MCI-S with respect to age and gender, both having previously been associated with morphometric measures. Thus, and in order to prevent the use of a high number of covariates in statistical analyses (and the risk to further reduce statistical power), control subjects were recruited for group-wise matching to each patient group with respect to these potentially confounding factors.

Additionally, the comparison of the data between MCI-S and MCI-CB, and between MCI-CB and MCI-CC would be of high interest. However, and apart from the cross-sectional nature of the present study, these analyses require accounting for confounding variables (i.e., variable times to conversion), and increase the number of comparisons. Generally speaking, and in consideration of the rather low sample sizes in the present study, additional analyses would further reduce the power of the present results. Similarly, ROC analyses discriminating MCI-CB from MCI-SB based on striatal and thalamic shape alterations is relevant. However, and for similar reasons and consistency, the main aim of the ROC analyses in the present study was to demonstrate similar if not improved abilities of striatal / thalamic shape alterations compared to hippocampal volumes for AD to discriminate between MCI-CB from HC. But first and foremost, the aim of the present study was to investigate morphometric differences between patient and well matched control groups to establish the sophisticated analysis techniques on images obtained from a 1.5 Tesla scanner. Nevertheless, further studies are now required to confirm our results in larger samples allowing for additional and more advanced statistical models.

MCI-S has been described as a potential early stage of AD (Bossa et al., 2011). However, apart from slightly reduced striatal volumes, MCI-S demonstrated no morphometric changes when compared to HC in the present study, rendering this notion unlikely. At the same time, MCI-S, consisting of amnestic MCI multiple domain subjects only, showed a more widespread pattern of cognitive impairment than MCI-CB, consisting of both, amnestic MCI single domain (*n* = 3) and amnestic MCI multiple domain subjects (*n* = 7). More precisely, both MCI groups showed memory impairment and impairment in a language-related function (confrontation naming) as well as in semantic memory and executive functioning (animal fluency). Interestingly, however, MCI-S but not MCI-CB showed further impairments in additional executive tasks (letter fluency, digit span backwards) as well as in attentional abilities (TMT A and TMT B). These attentional deficits in MCI-S might be at the basis of the other cognitive deficits in this group, resulting in a more widespread and global pattern of cognitive impairment when compared with MCI-CB–with remaining abilities for everyday life at the same time. Generally speaking, these findings might represent non-typical-AD pathology rather than early stage AD in the present MCI-S group, and this assumption is further supported by the morphometric findings in this group (reduced striatal volumes, no other morphometric abnormalities). However, the AD typical cognitive impairment, the pattern of cortical thinning and hippocampal atrophy in MCI-CB and MCI-CC, together with a cognitive profile uncharacteristic for AD and the absence of morphometric alterations in MCI-S, is supportive of AD pathology in MCI-CB and MCI-CC. Additional markers of neurodegeneration such as CSF levels of phosphorylated Tau together with amyloid imaging may provide additional information on this matter in future longitudinal studies. Also, vascular risk factors such as hypertension, diabetes mellitus, hyperlipidemia, etc. represent main risk factors for the development of dementia. This information, however, was not available for all subjects and could therefore not be added in the statistical models.

The similarity of patterns of morphometric alterations in MCI-CB and MCI-CC is most likely due to both groups consisting of the same subjects, with MCI-CC data having been obtained at time of conversion. Hence, the time lag of approximately 20.8 months between MRI at baseline and at time of conversion might have been too short to reveal progression of morphometric alterations.

## Conclusion

In conclusion, the simultaneous presence of highly accurate thalamic and striatal shape alterations, AD typical cortical thinning and hippocampal atrophy in MCI-CB but not in MCI-S highlights the value of subcortical shape alterations as early marker for AD, and emphasizes the importance to consider regional morphological information of subcortical structures. It is necessary to find ways allowing the implementation of advanced segmentation and analysis techniques in everyday clinical practice in the near future.

## Ethics statement

The study protocol was approved by the cantonal ethics committee Zurich, Switzerland, and the study was carried out in accordance with good clinical practice guidelines issued by the committee. All subjects gave written informed consent in accordance with the Declaration of Helsinki.

## Author contributions

Research questions and study design were formulated by SL, RN, AG, CH, and AK. Data acquisition was carried out by AK, LM, CS, SB, SK, AG, and SL. Data analyses were performed by AK, MP, MC, JL, LM, CS, and SL. All authors contributed to the interpretation of data and to the manuscript drafting, writing and revising.

## Funding

This study was made possible by SNF SPUM Grant No. 33CM30-124111, by the Molecular Imaging Network Zürich (MINZ), and by an International Short Visits Grant from the SNF to SL (IZK0Z3_151124). MC is funded by the Canadian Institutes of Health Research, National Sciences and Engineering Research Council of Cananda, Weston Brain Institute, Alzheimer's Society, and the Michael J. Fox Foundation for Parkinson's Research. RN and CH are founder/co-founder and board members of Neurimmune Holding AG, Schlieren.

### Conflict of interest statement

The authors declare that the research was conducted in the absence of any commercial or financial relationships that could be construed as a potential conflict of interest. RN and CH are founder/co-founder and board members of Neurimmune Holding AG, Schlieren.

## Abbreviations

CA: Cornu ammonis
eTIV: estimated total intracranial volume
HC: Healthy controls
ICBM: International consortium for brain mapping
ICV: intracranial volume
INSECT: Intensity-Normalized Stereotaxic Environment for Classification of Tissues
LGN: lateral geniculate nucleus
MAGeT: Multiple automatically generated templates
MCI-CB: mild cognitive impairment to AD converters at baseline
MCI-CC: mild cognitive impairment converters to AD at time of conversion
MCI-S: stable mild cognitive impairment at baseline
MGN: medial geniculate nucleus
NINCDS-ADRDA: National Institute of Neurological and Communicative Disorders and Stroke and the Alzheimer's Disease and Related Disorders Association
RMINC: R for Medical Imaging NetCDF
VA: ventral anterior nucleus
VL: ventral lateral nucleus
VP: ventral posterior nucleus.
